# Rapid and Inexpensive Method of Loading Fluorescent Dye into Pollen Tubes and Root Hairs

**DOI:** 10.1371/journal.pone.0152320

**Published:** 2016-04-07

**Authors:** Haiyong Qu, Wenxi Xing, Fenfen Wu, Yongzhang Wang

**Affiliations:** 1 College of Horticulture, Qingdao Agricultural University, No. 700 Changcheng Road, Chengyang, Qingdao City, Shandong Province, China; 2 Department of Horticulture, Qingdao Agricultural University, China, Qingdao City, China; Cinvestav-IPN, MEXICO

## Abstract

The most direct technique for studying calcium, which is an essential element for pollen tube growth, is Ca^2+^ imaging. Because membranes are relatively impermeable, the loading of fluorescent Ca^2+^ probes into plant cells is a challenging task. Thus, we have developed a new method of loading fluo-4 acetoxymethyl ester into cells that uses a cell lysis solution to improve the introduction of this fluorescent dye into pollen tubes. Using this method, the loading times were reduced to 15 min. Furthermore, loading did not have to be performed at low (4°C) temperatures and was successful at room temperature, and pluronic F-127 was not required, which would theoretically allow for the loading of an unlimited number of cells. Moreover, the method can also be used to fluorescently stain root hairs.

## Introduction

Pollen tube growth is crucial for the delivery of sperm cells to the ovule during angiosperm reproduction. Brewbaker and Kwack (1963) [1] extensively investigated the relationship between Ca^2+^ concentrations ([Ca^2+^]) and pollen tube growth under *in vitro* germination culture conditions, and they demonstrated that Ca^2+^ is essential for *in vitro* pollen tube growth; and Iwano et al. (2004) [2] showed that submicromolar [Ca^2+^]_i_ in the tip region is necessary for pollen tube growth in the style. However, the role of intracellular Ca^2+^ can only be investigated by monitoring its concentration dynamics in plant cells with both spatial and temporal accuracy [3]. Because critical measurements of [Ca^2+^]_i_ are essential for assessing transduction pathways and downstream events in tip-growing cells, the applied imaging method is also important. Consequently, various Ca^2+^ probes for Ca^2+^ imaging have been developed over the past six decades.

Genetically encoded calcium sensors can efficiently measure calcium concentrations in cellular organelles, especially in *in vitro* or *in vivo* single cells. Furthermore, calcium sensors can be used in transformation and cell biology to study sexual organs that are accessible and easily visualized and handled [4], such as those of *Nicotiana tabacum* [5] and *Arabidopsis thaliana* [2]. In certain plants the construction of a stable transgenic system is difficult; however, the use of small, fluorescent, calcium-sensing molecules has been enormously beneficial for the spatiotemporal mapping of intracellular calcium signals in these plants. The loading of Ca^2+^-sensitive fluorescent dyes into cells is difficult because of the plasma membrane, which is a protein-lipid bilayer that forms a closed bimolecular sheet separating the cell contents from the extracellular environment. Ca^2+^-sensitive dyes have been used in a variety of forms (as free acids or acetoxymethyl [AM] esters or in conjugation with high molecular weight dextrans) and loaded using different methods, including ester diffusion, ionophoresis and pressure micro-injection. Of all the tested methods, pressure (hydraulic) micro-injection of fluorescent dyes is the most difficult to perform, although it provides the best measurements for the longest period of time (>2 h) [6]. Therefore, several researchers have adopted this method for fluorescent dye loading into growing pollen tubes to measure [Ca^2+^]_i_ [7,8]. Because micro-injection is a mechanical method of opening the cell membrane and enabling fluorescent dye penetration into cells, we speculated that cell lysis could be applied as an alternative approach. Cell lysis dissolves the phospholipid bilayer of cell membranes by forming water-soluble complexes with their constituents, and it aids in the extraction of cellular proteins, DNA and other cell contents. Compared with the amount of cell lysis solution required for DNA extraction, the quantity needed to facilitate dyeing is much lower. In this study, we investigated the use of cell lysis-aided loading of fluorescent dyes into pollen tubes.

## Materials and Methods

### Pollen collection and culturing

Pollen grains of *Pyrus pyrifolia* were collected from an experimental fruit orchard in Jiangsu Province, China. The pollen grains were collected from harvested blooming flowers and preserved at −20°C until culturing. The experimental pear orchard is a state-owned experimental field located in Gaoyou City, Jiangsu Province, China. Any individuals or research group can freely obtain pollen and branches for experimental use only (not for commercial purposes). The pollen culture medium contained 1.0 mM KCl, 0.06 mM CaCl_2_, 1.0 mM H_3_BO_3_ and 1.0 mM MES (2-(N-morpholino)ethanesulfonic acid) at pH 6.3 (Tris).

### Intracellular calcium detection

Fluo-4 acetoxymethyl ester (fluo-4/AM; Dojindo Laboratories, Kumamoto, Japan) was used as a fluorescent indicator to detect intracellular calcium in the pollen. A combination of tetra (AM) ester (C_51_H_50_F_2_N_2_O_23_ = 1096.94) and 1-[2-amino-5-(2, 7-difluoro-6-acetoxymethoxy-3-oxo-9-xanthenyl) phenoxy]-2-(2-amino-5-methylphenoxy) ethane-N,N,Nʹ,Nʹ-tetraacetic acid was used as a fluorescent chelator. This combination is excited by visible light at ~494 nm and emits a yellowish green fluorescence at ~516 nm when bound to calcium ions. The cell lysis solution contained 2% (W/V) cetyltrimethylammonium bromide (CTAB), 100 mM Tris-HCl and 40 mM EDTA at pH 8.0.

Pollen tube fluorescence was developed and detected using the following steps:

1. Exactly 50 μg of fluo-4/AM was dissolved in 91.2 μL of anhydrous dimethylsulfoxide (Dojindo) in a microcentrifuge tube. Immediately prior to use, 100 μM of the fluo-4/AM working solution was prepared by diluting the fluo-4/AM solution with culture medium.
2. The cell lysis solution was diluted 10-fold with ddH_2_O.
3. The pollen grains were suspended in the culture medium at a concentration of 1 × 10^6^ cells/mL and cultured at room temperature (25°C) for 2 h. Then, 48 μL of this culture was added to individual microcentrifuge tubes.
4. Exactly 1 μL of the fluo-4/AM/AM mixture (from step 1) and 1 μL of the cell lysis solution (step 2) were added to the centrifuge tubes containing the pollen tube culture (step 3). The tubes were left to react for 15 min.
5. After fluo-4/AM loading has completed, the pollen tubes were washed three times with dye-free culture medium (1 mL). The pollen tube fluorescence was then detected using an Olympus IX73 inverted microscope (Shinjuku-ku, Tokyo, Japan).

### Influence of Cd^2+^, La^3+^ and EGTA on fluorescence

Cd^2+^ and La^3+^ are widely used to affect intracellular Ca^2+^ concentrations in both animal and plant cells. After step 3 of the protocol, Cd^2+^ at a final concentration of 50 μM or La^3+^ at a final concentration of 10, 100 and 1000 μM was added to the culture medium. Pollen tubes growing under normal conditions after loading fluo-4/AM without Cd^2+^ or La^3+^ was used as a control. EGTA (ethylene glycol bis (2-aminoethyl ether)-N, N, N’, N’-tetraacetic acid) at a final concentration of 1 mM was added to the medium.

### Arabidopsis and tobacco pollen tubes dyed with fluo-4/AM

Cultivation of Arabidopsis (*Arabidopsis thaliana*) plants was performed according to Iwano et al. (2004) [2]. The light intensity was 120–150 lmol m^-2^ s^-1^ over a 12-h daily light period. The temperature was 22 ± 2°C.

*Arabidopsis thaliana* flowers that were newly open were picked, and pollen grains were dusted on the medium. The pollen was cultured for 6 h at 25°C in medium containing 18% (w/v) sucrose, 0.01% (w/v) H_3_BO_3_, 1 mM CaCl_2_, 1 mM Ca(NO_3_)_2_, and 1 mM MgSO_4_ at pH 7.0 (KOH). *Arabidopsis thaliana* root hairs were cultured for 4 weeks. The tobacco pollen was cultured for 3 h at 25°C in medium that contained 5 mM H_3_BO_3_, 8 mM MgSO_4_.7H_2_O, 1 mM KCl, 5 mM CaCl_2_, 10 mM inositol, 5 mM MES, and 20% sucrose at pH 7.0 (KOH). The methods of loading fluo-4/AM into the pollen tubes and root hairs of Arabidopsis or tobacco were same as those used for the *P*. *pyrifolia* pollen tube.

### Image analysis

The fluorescence results were analyzed using Image-Pro Plus software (S1 Fig and S2 Fig) and Microsoft Excel 2007. The pseudo colors in the figures were added using the software Confocal Assistant 4.02 (University of Minnesota, USA). For the final processing, we used Adobe Photoshop CS5 (Adobe Systems, Mountain View, CA).

## Results

### Cell lysis solution promoted fluo-4/AM loading into pollen tubes

**Fig 1:**
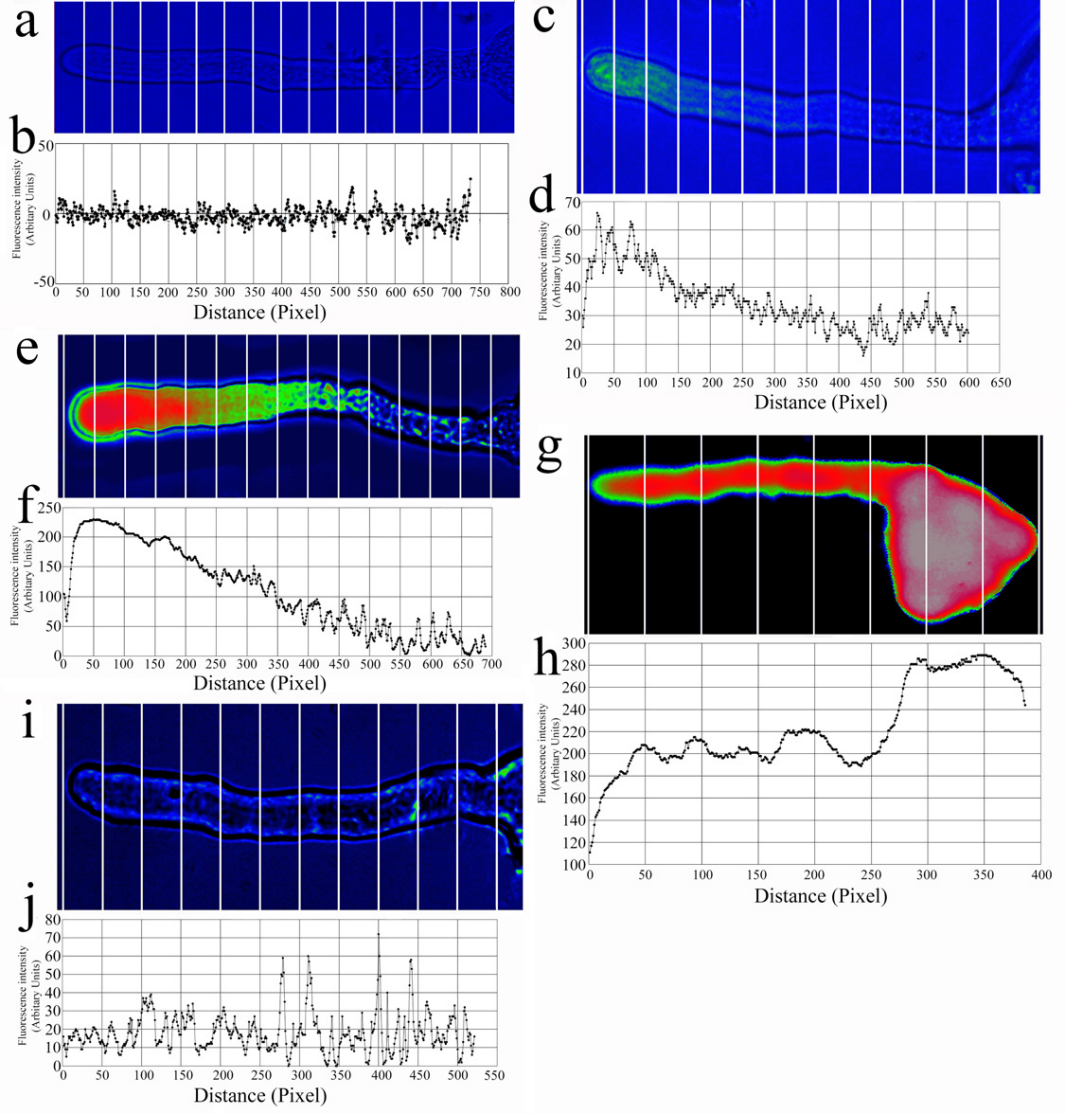
Loading of fluo-4/AM into pollen tubes under different conditions. (a, b) Control pollen tube that was not loaded with fluo-4/AM. (c-h) Pollen tubes loaded with fluo-4/AM along with cell lysis solution for 5 min (c, d), 15 min (e, f) and 30 min (g, h). (i, j) Pollen tubes loaded for 30 min without cell lysis solution.

The pollen tubes did not display fluorescence (Fig 1A and 1B) prior to loading with fluo-4/AM. After loading with the cell lysis solution containing fluo-4/AM (Fig 1C and 1D) for 5 min, the Ca^2+^ gradient from the tip to the base of the pollen tubes was not particularly apparent because the fluorescence was weak. After loading for 15 min, the Ca^2+^ gradient was pronounced and the fluorescence signal was evident across the entire pollen tube, especially at the tip (Fig 1E and 1F). When the cell lysis solution reached a loading time of 30 min (Fig 1G and 1H), the Ca^2+^ gradient disappeared. In addition, the pollen tubes that were not exposed to cell lysis solution (the controls) showed no fluorescence at a loading time of 30 min, although the cell walls and membranes did produce a weak fluorescence (Fig 1I and 1J). These results suggest that the cell lysis solution can promote fluo-4/AM loading into pollen tubes.

### Comparison of the influence of the cell lysis solution and pluronic F-127 on fluo-4/AM loading

**Fig 2:**
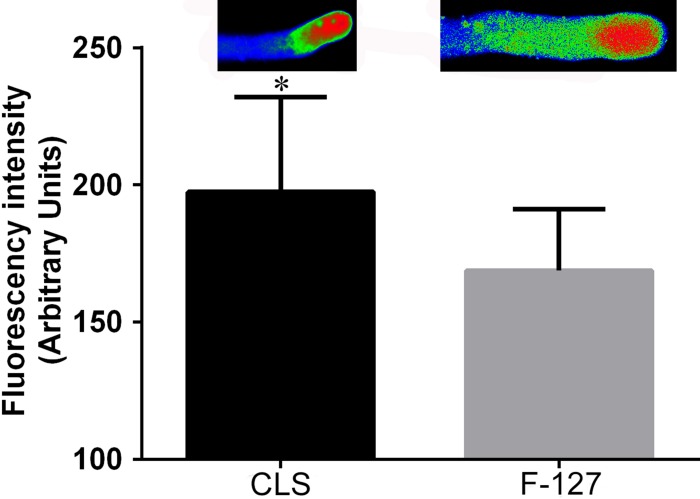
Comparison of the influence of the cell lysis solution and pluronic F-127 on fluo-4/AM dyeing. Fluorescence intensity using cell lysis solution as an auxiliary reagent was significantly higher (*P* < 0.05, Student’s t-test) compared with the values obtained using pluronic F-127. Vertical bars indicate ± SE. Each data point represents the mean of three replicates with more than 10 pollen tubes each. CLS: cell lysis solution.

To further demonstrate the ability of cell lysis solution to enhance the loading of fluo-4/AM into pollen tubes, we loaded fluo-4/AM into pollen tubes along with pluronic F-127 at 4°C for 2 h. The resulting fluorescence intensity at the apical pollen tube region was lower than that obtained using the cell lysis solution auxiliary method (Fig 2).

**Fig 3:**
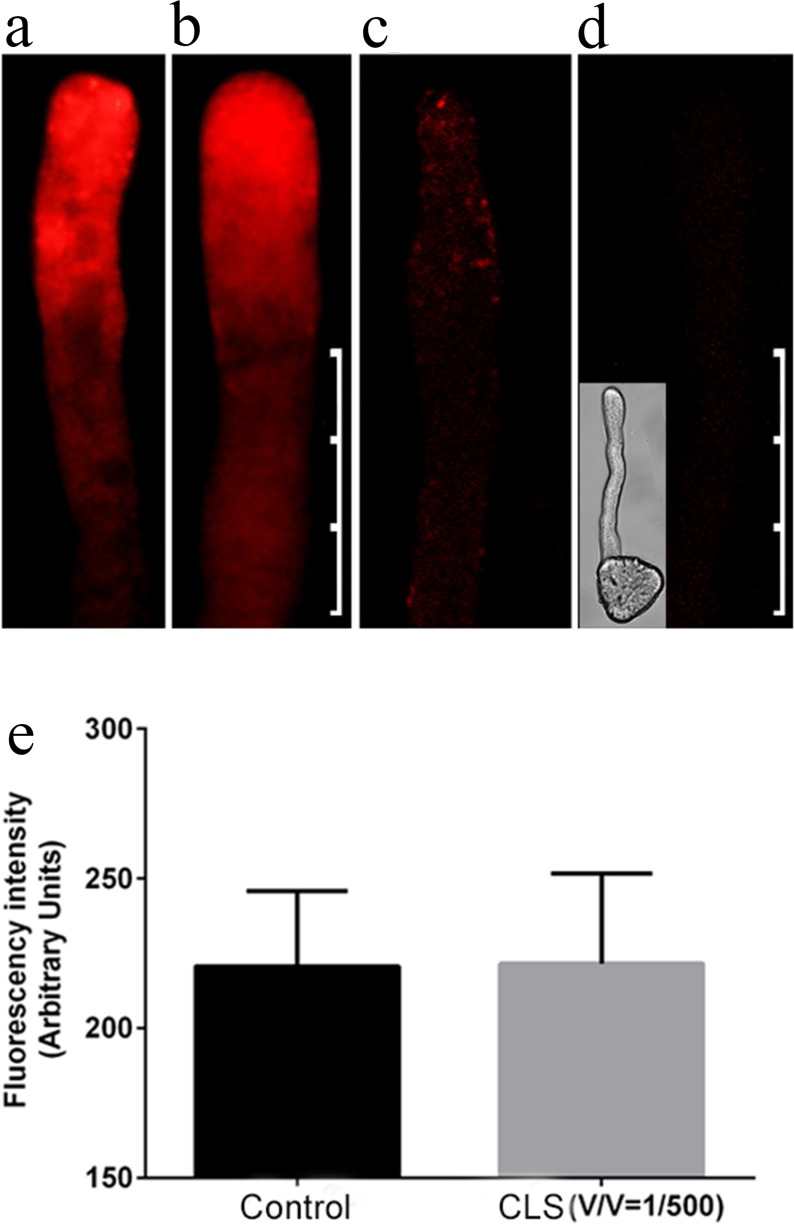
Effect of the cell lysis solution on the pollen tube membranes. (a) Pollen tube dyed with FM4-64 without cell lysis solution (control). (b and d) Pollen tubes after dyeing with FM4-64 for 15 min with 1:500 (v/v) cell lysis solution in the medium (b), with 1:5 (v/v) cell lysis solution in the medium (c) and with 1:1 (v/v) cell lysis solution in the medium (d). (e) Fluorescence value comparison. The difference was not significant. Scale bars: 50 μm (a and b) and 60 μm (c and d). Vertical bars indicate ±SE. Each data point represents the mean of three replicates with more than 10 pollen tubes each.

After treatment for 10 min with the cell lysis solution at the same concentration used for auxiliary dyeing (1:500 [v/v] dilution), the pollen tubes were dyed with FM4-64 for 15 min. Differences were not observed between this treatment and the control (Fig 3A, 3B and 3E), and similar results were found for the pollen tubes of *Picea meyeri* Rehd. Et Wils and *Picea wilsonii* Mast [9,10]. However, as the cell lysate concentration in the medium increased, the fluorescence became progressively weaker. When the volume ratio of lysate to medium reached 1:1, the pollen tubes exhibited little fluorescence (Fig 3C and 3D). This result implies that the cell lysis buffer has no effect on the cell membranes at low concentrations but can damage cell membranes at high concentrations.

### Cd^2+^, La^3+^ and EGTA affect the Ca^2+^ gradient

**Fig 4:**
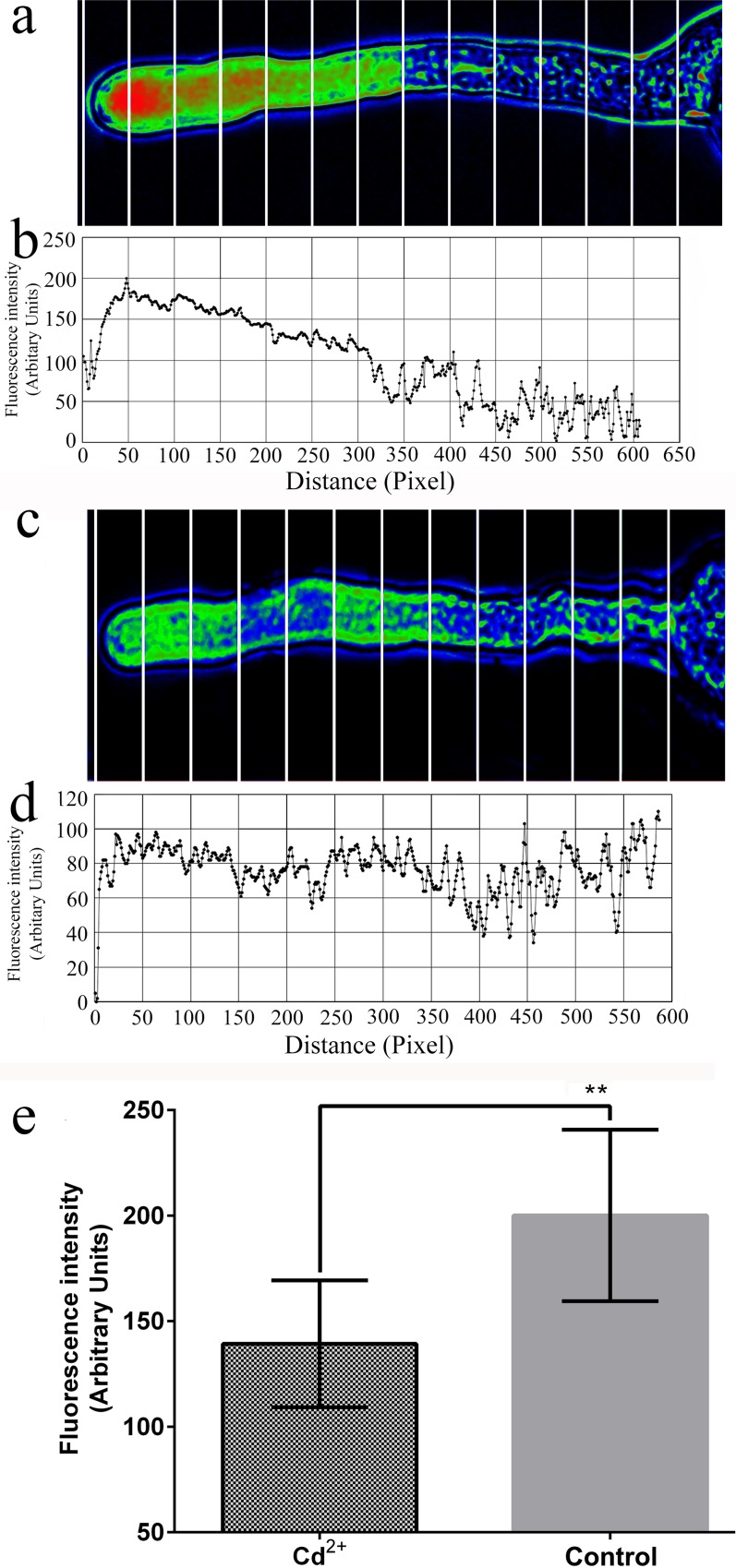
Effect of Cd^2+^ on the Ca^2+^ gradient in pollen tube apical regions. (a, b) Results obtained using a loading time of 15 min and the cell lysis solution (control). (c, d) Results after inclusion of 50 μM Cd^2+^ during loading. (e) Statistical analysis of fluorescence values at the pollen tube tips (*n* = 15). ** corresponds to a significant difference (*P* < 0.01, Student’s t-test). Vertical bars indicate ± SE. Each data point represents the mean of three replicates with more than 10 pollen tubes each. CLS: cell lysis solution.

**Fig 5:**
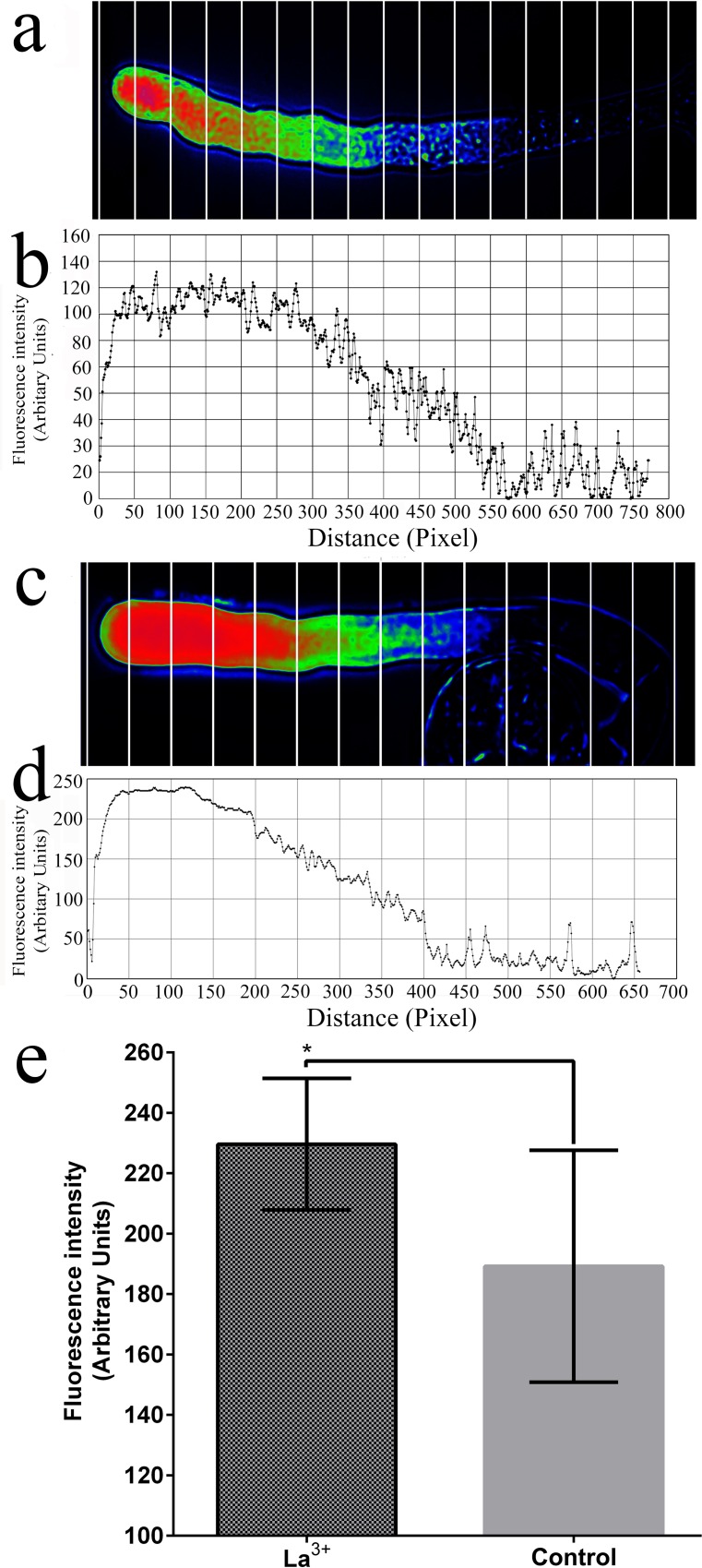
Effect of La^3+^ on the Ca^2+^ gradient in the pollen tube apical regions. (a, b) Results obtained using a loading time of 15 min and the cell lysis solution (control). (c, d) Results after inclusion of 10 μM La^3+^ during loading. (e) Statistical analysis of fluorescence values at the pollen tube tips (*n* = 10). * corresponds to a significant difference (*P* < 0.05, Student’s *t*-test). Vertical bars indicate ±SE. Each data point represents the mean of three replicates with more than 10 pollen tubes each.

**Fig 6:**
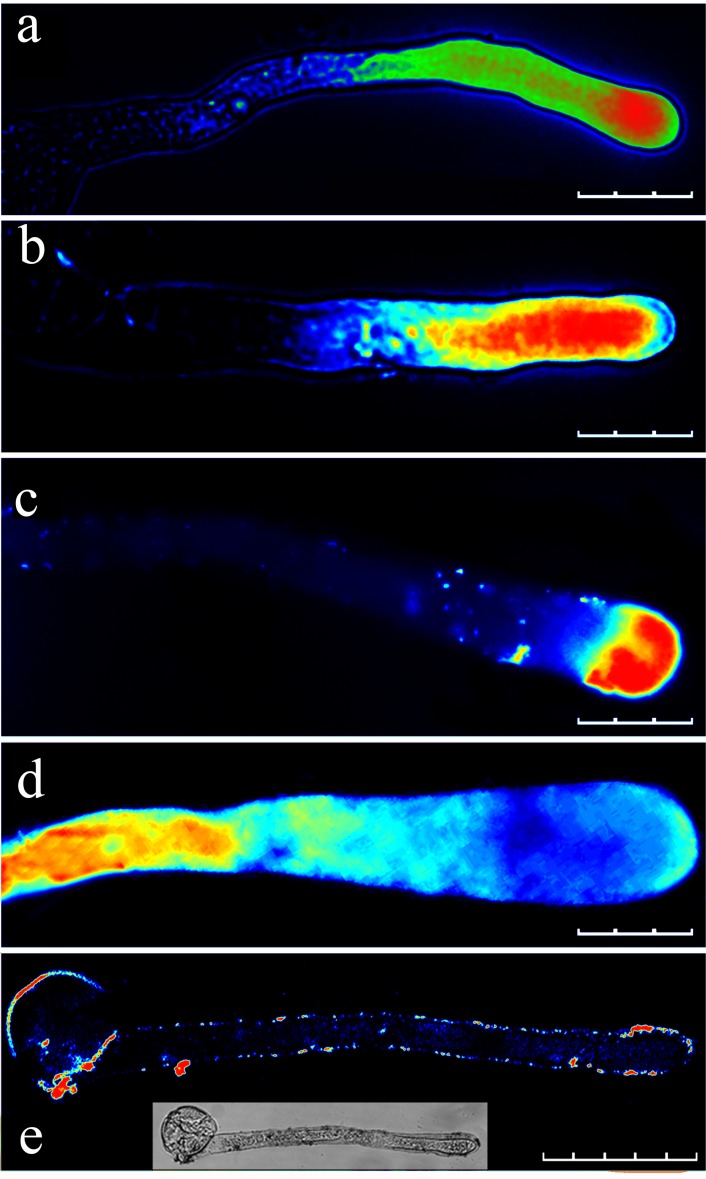
Effect of different concentrations of La^3+^ on the calcium gradient in the pollen tube apical regions. (a) Pollen tube loaded with fluo-4/AM without La^3+^ (control). Pollen tubes after loading with fluo-4/AM followed by the addition of 10 μM La^3+^ (b), 100 μM La^3+^ (c), and 1 mM La^3+^ (d). (e) Pollen tube after loading with fluo-4/AM in the presence of 1 mM La^3+^. Scale bars: 100 μm (a), 90 μm (b and c), 60 μm (d) and 70 μm (e).

**Fig 7:**
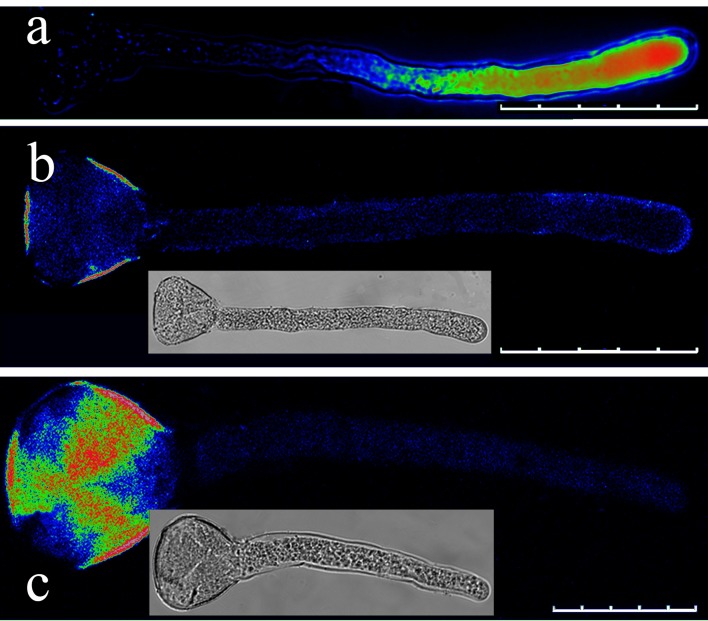
Effect of EGTA on the Ca^2+^ gradient in the pollen tube apical regions. (a) Pollen tube loaded with fluo-4/AM without EGTA (control). (b) Pollen tube after addition of 1 mM EGTA following fluo-4/AM loading. (c) Pollen tube after addition of 1 mM EGTA during fluo-4/AM loading. Scale bars: 100 μm (a), 60 μm (b) and 30 μm (c).

During the *in vitro* pollen culture, pollen tube growth was arrested by inhibiting Ca^2+^ channels on the membranes or removing extracellular Ca^2+^. According to our study results, Cd^2+^ can inhibit Ca^2+^ channels in the apical regions of *P*. *pyrifolia* pollen tubes (S3 Fig). After applying the 50 μM Cd^2+^ treatment for 10 min, the Ca^2+^ gradient disappeared in the pollen tube (Fig 4A–4D). In addition, the fluorescence at the pollen tube tips was significantly lower after the Cd^2+^ treatment compared with that in the control (Fig 4E). However, the 10 μM La^3+^ treatment produced only slight effects on the Ca^2+^ gradient (Fig 5A–5D and Fig 6A and 6B), although after the La^3+^ treatment, the fluorescence at the pollen tube tips was significantly higher compared with that in the control (Fig 5E). Moreover, the 100 μM La^3+^ treatment did not have an effect on the Ca^2+^ gradient (Fig 6A or 6C), whereas at 1 mM, the entire pollen tube displayed fluorescence and the Ca^2+^ gradient had disappeared from the tip of the pollen tube (Fig 6D). An interesting result was obtained when we added 1 mM La^3+^ to the medium while loading the fluo-4/AM dye: the Ca^2+^ gradient disappeared and fluorescence was weak in the pollen tube and mainly concentrated in the pollen tube wall (Fig 6E). This result suggests that La^3+^ inhibited the activity of Ca^2+^ channels on the membrane and decreased the influx of calcium. When the calcium-selective chelator EGTA (1 mM) was added to the medium during or after fluo-4/AM loading, the Ca^2+^ gradient at the apical pollen tube region was disrupted (Fig 7A–7C). This finding indicates that the removal of extracellular Ca^2+^ by EGTA led to the disintegration of the Ca^2+^ gradient at the pollen tube tip.

### Cell lysis solution promoted fluo-4/AM loading into the root hair and pollen tubes of Arabidopsis

**Fig 8:**
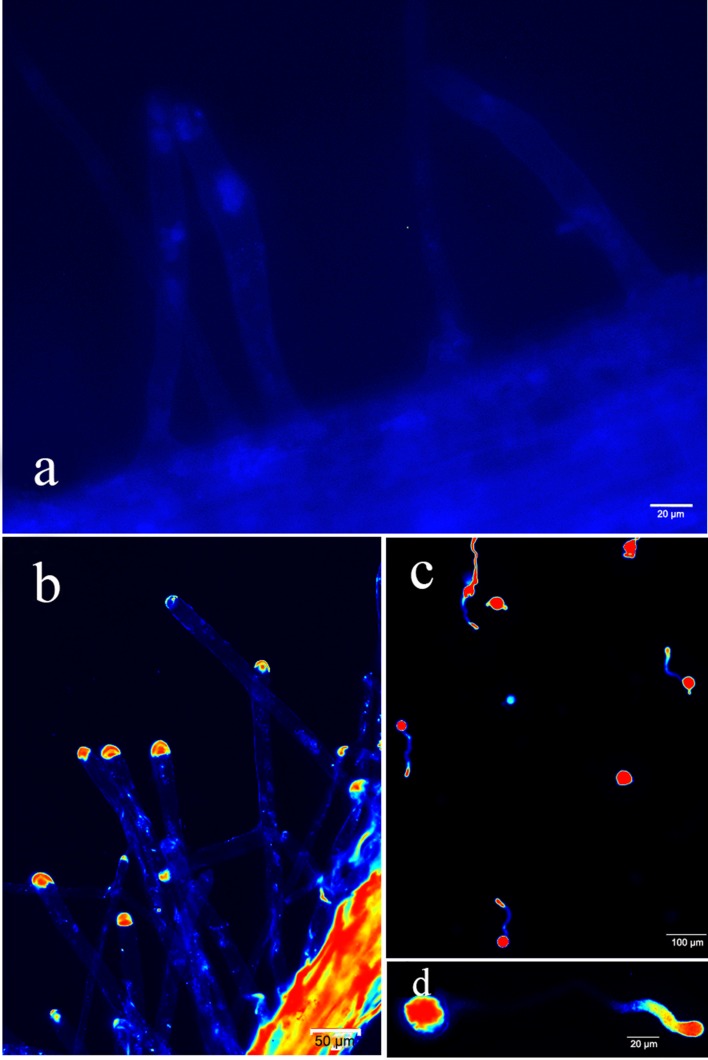
Loading fluo-4/AM into the root hair and pollen tube of *Arabidopsis*. (a) Control root hair that was not loaded with fluo-4/AM. (b) Root hair loaded with fluo-4/AM along with cell lysis solution for 15 min. (c-d) Pollen tubes loaded with fluo-4/AM along with cell lysis solution for 15 min. Note: The root hairs were barely observable in the fluorescence microscopy image because the autofluorescence of the root hairs was very weak. To see the root hairs clearly in the image, we improved the overall image brightness with Photoshop software. Therefore, the background of image (a) is different from the background of the other images (b, c and d).

To further verify this method, we loaded fluo-4/AM into the root hair and pollen tubes of Arabidopsis. The root hair of Arabidopsis did not display fluorescence (Fig 8A), and the Ca^2+^ gradient distribution in the root hair was different from the distribution in the pollen tubes of *P*. *pyrifolia*. The concentration of Ca^2+^ mainly concentrated in the root hair tip, and almost no Ca^2+^ was distributed in the sub-tip (Fig 8B). The Ca^2+^ gradient distribution in the pollen tubes of Arabidopsis was equivalent to that in the pollen tubes of *P*. *pyrifolia* (Fig 8C and 8D). The Ca^2+^ concentration at the apical pollen tube fluctuated during pollen tube growth (S4A and S4B Fig and S1 Video). The pollen tube length also presented fluctuations every two minutes (S4C Fig). Two types of oscillation were relatively consistent. The resulting Ca^2+^ concentration at the tip of the pollen tube was closely related to the pollen tube growth, and this result was consistent with the relationship between pollen tube growth in *Lilium longiflorum* and intracellular Ca^2+^ concentration oscillations [11,12]. We also successfully loaded fluo-4/AM into the pollen tube of *Nicotiana tabacum* L. (S5 Fig), and the results suggested that the dyeing method could be applied to plant root hairs and pollen tubes of both of the studied plants.

## Discussion

Although Ca^2+^-sensitive fluorescent indicators are among the most powerful tools for quantifying the spatial and temporal dynamics of [Ca^2+^]_i_, loading probes into living plant cells is a challenging task. Because most plant cell membranes are impermeable, only a few plant cell types can be loaded directly with Ca^2+^ indicators. Takahashi et al. (1999) [6] reviewed 12 dye-loading procedures and found that each method has advantages and disadvantages with respect to measuring intracellular Ca^2+^ concentrations. For example, micro-injection is advantageous because the charged form of the indicator can be directly loaded into the cytosol with certainty; however, this method requires specialized instrumentation and practice. In addition, only a limited number of cells can be loaded with the probes. Many fluorescent Ca^2+^ indicators are combined with a cell-permeable AM ester. However, to avoid cell-wall hydrolysis of the ester of fluo-4/AM by cell-wall esterase, the loading must occur at low temperatures [13]. As a result, the loading requires a relatively long time to complete; for instance, loading fluo-4/AM into pear pollen tubes requires 1 h [14], loading fluo-3/AM into lily pollen tubes requires 2 h [15,16] and loading fluo-3 into rice root hairs requires 4 h [13]. Other examples include the loading of Fura-2/AM into rabbit heart cells [17] and *Salmonella enterica* cells [18], which require 90 and 75 min, respectively. A major problem associated with such extended loading times is that the loading dye is readily compartmentalized inside cells. Gee et al. (2000) [19] suggested that this compartmentalization might be avoided by reducing the loading time.

A cell lysis solution is a detergent-based buffer solution used to break open target cells and further isolate a particular cellular component of interest. Therefore, we used this reagent to disintegrate the lipid bilayer. We found that only a small volume of cell lysis solution (1:500 volume ratio) was needed to achieve this disruption, which also occurred rapidly (15 min). The reagent degraded the membrane structure slightly, which allowed fluo-4/AM to enter the pollen tube more efficiently. According to the FM4-64 fluorescent indicator measurements, the pollen tube membrane was undamaged after the cell lysis (1:500 volume ratio) treatment. Furthermore, pollen tube activity was hardly affected (S4 Fig, S1 Video and S6 Fig). However, cell lysis damages the cell membrane; therefore, when cell lysis auxiliary loaded fluorescent reagents are applied, the suitable cell lysis concentration and dyeing time must be used or else the process will damage the cell membrane and cause a loss of cell viability (Fig 1G and Fig 3D).

Although pluronic F-127 has been used to facilitate the dispersal of AM esters of fluorescent ion indicators, such as fura-2, indo-1, fluo-3 and fluo-4, this reagent has occasionally been found to decrease dye incorporation relative to the control. Pluronic F-127 thus apparently has some effect on cell membranes [20,21]. In addition, pluronic F-127 significantly alters depolarization-evoked [Ca^2+^]_cyt_ transients, which may reflect an alteration in the regulation of [Ca^2+^]_cyt_ in neuronal cells [22]. Considering these results, pluronic F-127 clearly should not be used to load fluorescence markers into single cells [20]. Our study findings indicate that the auxiliary loading effect of the cell lysis solution is superior to the effects of using pluronic F-127.

Cd^2+^ can inhibit Ca^2+^ influxes, thereby causing the Ca^2+^ gradient to disappear in the apical root hair cells [21]. The fluorescence intensity is also decreased by the disruption of actin filaments and Ca^2+^ gradients by CdCl_2_. Hinkle et al. (1992) [23] found that CdCl_2_ decreases the fluorescent intensity of pituitary GH_3_ cells through the replacement of Ca^2+^ by Cd^2+^. We conclude that CdCl_2_ was able to decrease fluorescence intensity in the pollen tube tips because Cd^2+^ inhibited the Ca^2+^ influx into the tubes.

La^3+^ has previously been shown to arrest pollen tube growth by inhibiting Ca^2+^ channels at the tip of the pollen tube [16]. Although Zhang et al. (2007) [24] demonstrated that La^3+^ at 10 or 100 μM can significantly change the morphology of germinated pollen tubes of *Lilium davidii* and destroy the Ca^2+^ gradient, we found that La^3+^ at 10 μM or 100 μM did not destroy the calcium gradient, and the concentration of 10 μM produced a considerable increase in the fluorescence intensity. The addition of a high concentration of La^3+^ (1 mM) to the medium caused the Ca^2+^ gradient to disappear. Previous research has also revealed that La^3+^ induces the release of an intracellular calcium pool into the cytoplasm [25,26]. If fluo-4/AM is restricted to the subcellular compartments, then La^3+^ would not increase the fluorescence intensity. Moreover, when measuring cytosolic Ca^2+^ responses, dye compartmentalization can be prevented by loading cells at room temperature [27]. Because our new dye-loading method is conducted at room temperature, we believe that compartmentalization will be avoided.

In conclusion, we used a cell lysis solution to aid in the penetration of fluorescent dye into pollen tube membranes. The addition of the cell lysis solution also eliminated the need for pluronic F-127. The 15-min room-temperature loading time was much shorter than what is typically required for loading at cool temperatures. Our method can be used with large numbers of cells. We successfully loaded fluo-4/AM into the root hair and pollen tubes of *Arabidopsis*, and we suggest that this method is suitable for other plant cell types.

## Supporting Information

S1 Fig Steps used to calculate the Ca^2+^ fluorescence intensity at the tip of the pollen tube. The calculation process is as follows:
**First step:** Use the software Image-Pro Plus to open the pollen tube fluorescence image, click on the "Measure" tool on the toolbar and then select "Profile Line" from the drop-down menu (S1a).**Second step:** Select the “Circle” in the “Line Profile” window and draw an ellipse at the tip of the pollen tube (S1b).**Third step:** Click the “File” in the “Line Profile” window and then click “Export data” from the drop-down menu (S1c).**Fourth step:** If the blank Excel form has already been opened, click "Data Export" to automatically import the data into the Excel form. Use the Excel function (AVERAGE) to calculate the average fluorescence intensity within the ellipse (S1d).Each treatment was repeated three times with more than 10 pollen tubes each.
(TIF)

S2 Fig Fluorescence density calculation steps are the same as in S1 Fig with the following changes: select “Freeform” from the “Line Profile” window and draw a curve from the tip to the base of the pollen tube. The calculation process is as follows:
Use the software Image-Pro Plus to open the pollen tube fluorescence image (S2a).Click on the "Measure" tool on the toolbar and then select “Freeform” from the “Line Profile” window (S2b).Select "File" and then click "Export" in the "Line Profile" window to export the data to Excel.
(TIF)

S3 Fig Inward currents recorded from a whole-cell patch clamp at the pollen tube apical region showing the effects of Cd^2+^ on the inward currents of the pollen tube apical spheroplast plasma membrane. Cd^2+^ is widely used as an effective Ca^2+^ channel blocker in both animal and plant cells. As shown in (a) and (b), 50 μM Cd^2+^ markedly inhibited hyperpolarization-activated inward currents (*n* = 5). (a) Normal Ca^2+^ currents recorded in 10 mM extracellular Ca^2+^ (control). The inset shows a pipette forming a giga seal with a protoplast membrane of the pollen tube apical region. Giga seal resistances were all greater than 1 GΩ. (b) Ca^2+^ currents with 50 μM Cd^2+^ added to the bath solution.
(TIF)

S4 Fig Images of *Arabidopsis* pollen tubes were collected every two minutes. Pollen tubes maintained Ca^2+^ gradients at the apex pollen tube for 15 min during normal growth (a-b). Ca^2+^ concentration fluctuation at the tip of the pollen tube during pollen tube growth. (c) Growth of the pollen tube length every two minutes. The intensity of the red color indicates higher concentrations of calcium ions.
(TIF)

S5 Fig Loading fluo-4/AM into the pollen tube of *Nicotiana tabacum* L. Pollen tubes loaded with fluo-4/AM along with cell lysis solution for 15 min.
(TIF)

S6 Fig Pollen was cultured at 25°C for 2 h, which was followed by the addition of cell lysis solution (1:500 volume ratio). After 15 min, the pollen was washed three times and the culturing was continued. No difference in pollen tube length was observed between the treatment and control at 3 h and 4 h. This result suggests that the pollen tube activity was unaffected by a brief (15 min) cell lysis solution treatment. More than 50 growing pollen tubes were quantified per line. Error bars represent ± SD. CLS: cell lysis solution.
(TIF)

S1 Video Serial images were converted to video format (S4A Fig) because such conversions can facilitate the viewing of changes in the Ca^2+^ concentration at the apical pollen tube during pollen tube growth. (MP4)
