# Supplementary figures and images for: Rapid and Inexpensive Method of Loading Fluorescent Dye into Pollen Tubes and Root Hairs
Source: PLoS One. 2016 Apr 7;11(4):e0152320. doi: 10.1371/journal.pone.0152320 (PMC4824429; doi:10.1371/journal.pone.0152320)

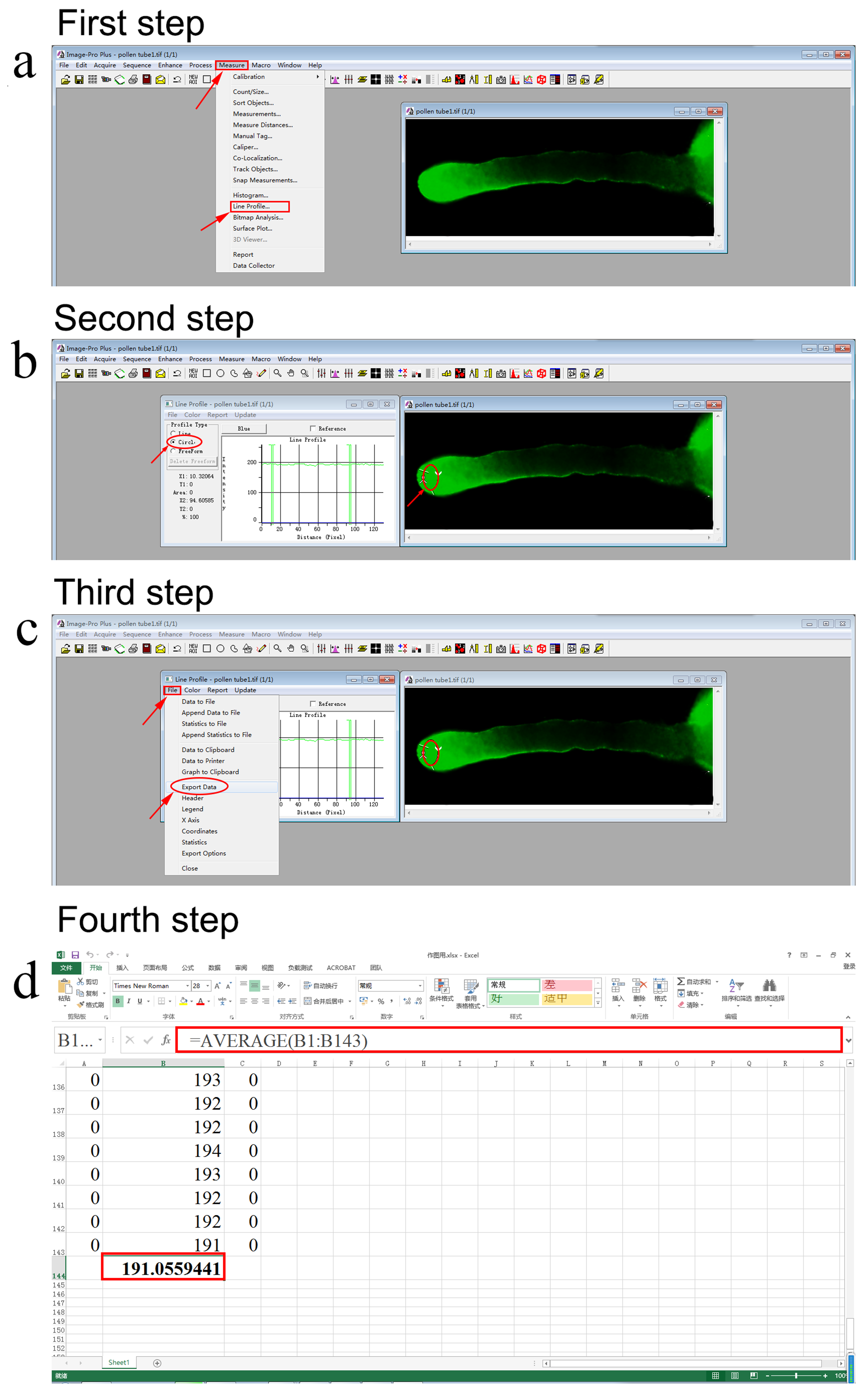

Supplement: S1 Fig — The calculation process is as follows: First step: Use the software Image-Pro Plus to open the pollen tube fluorescence image, click on the "Measure" tool on the toolbar and then select "Profile Line" from the drop-down menu (S1a).Second step: Select the “Circle” in the “Line Profile” window and draw an ellipse at the tip of the pollen tube (S1b).Third step: Click the “File” in the “Line Profile” window and then click “Export data” from the drop-down menu (S1c).Fourth step: If the blank Excel form has already been opened, click "Data Export" to automatically import the data into the Excel form. Use the Excel function (AVERAGE) to calculate the average fluorescence intensity within the ellipse (S1d).Each treatment was repeated three times with more than 10 pollen tubes each. (TIF) [file pone.0152320.s001.tif]

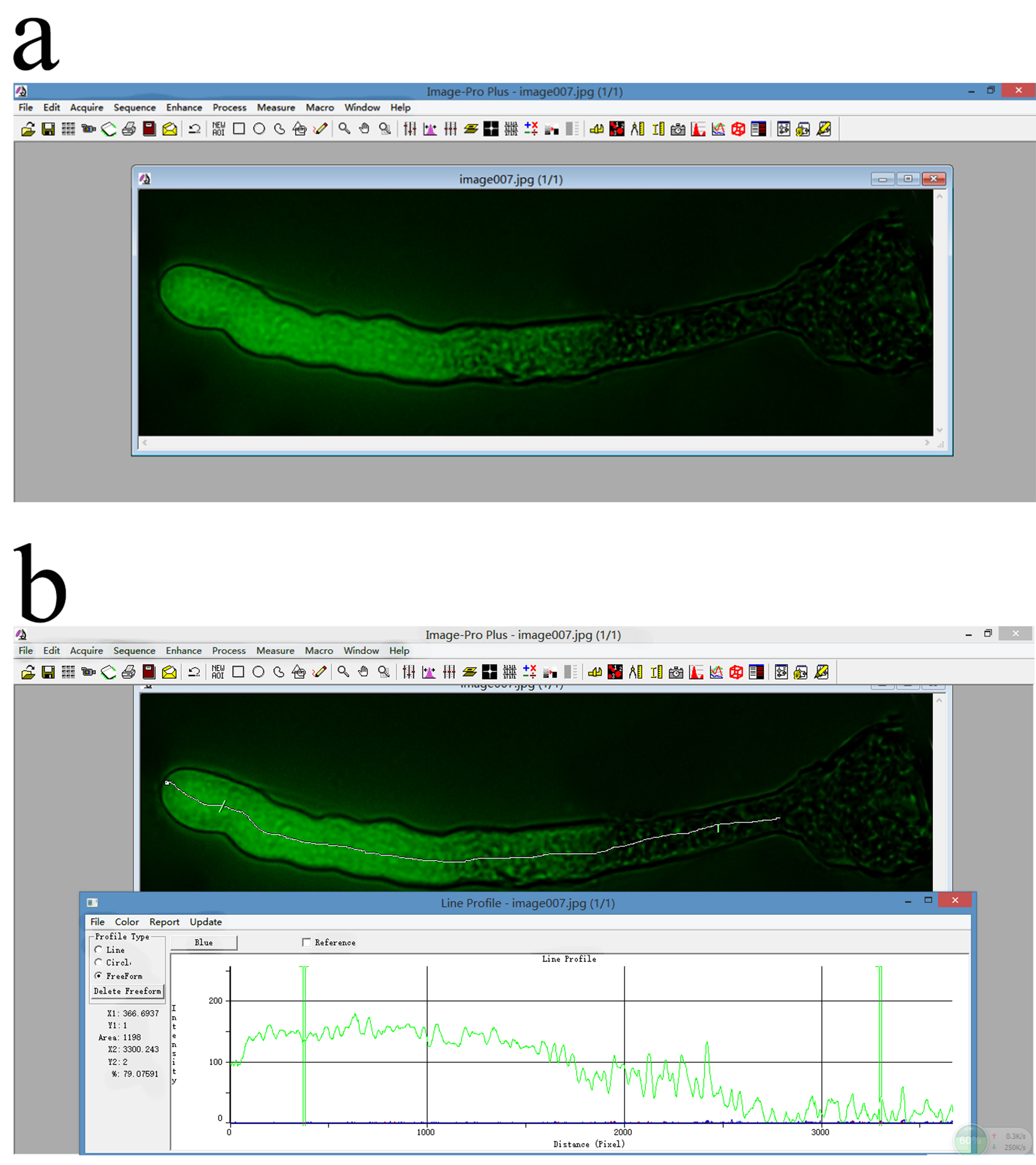

Supplement: S2 Fig — The calculation process is as follows: Use the software Image-Pro Plus to open the pollen tube fluorescence image (S2a).Click on the "Measure" tool on the toolbar and then select “Freeform” from the “Line Profile” window (S2b).Select "File" and then click "Export" in the "Line Profile" window to export the data to Excel. (TIF) [file pone.0152320.s002.tif]

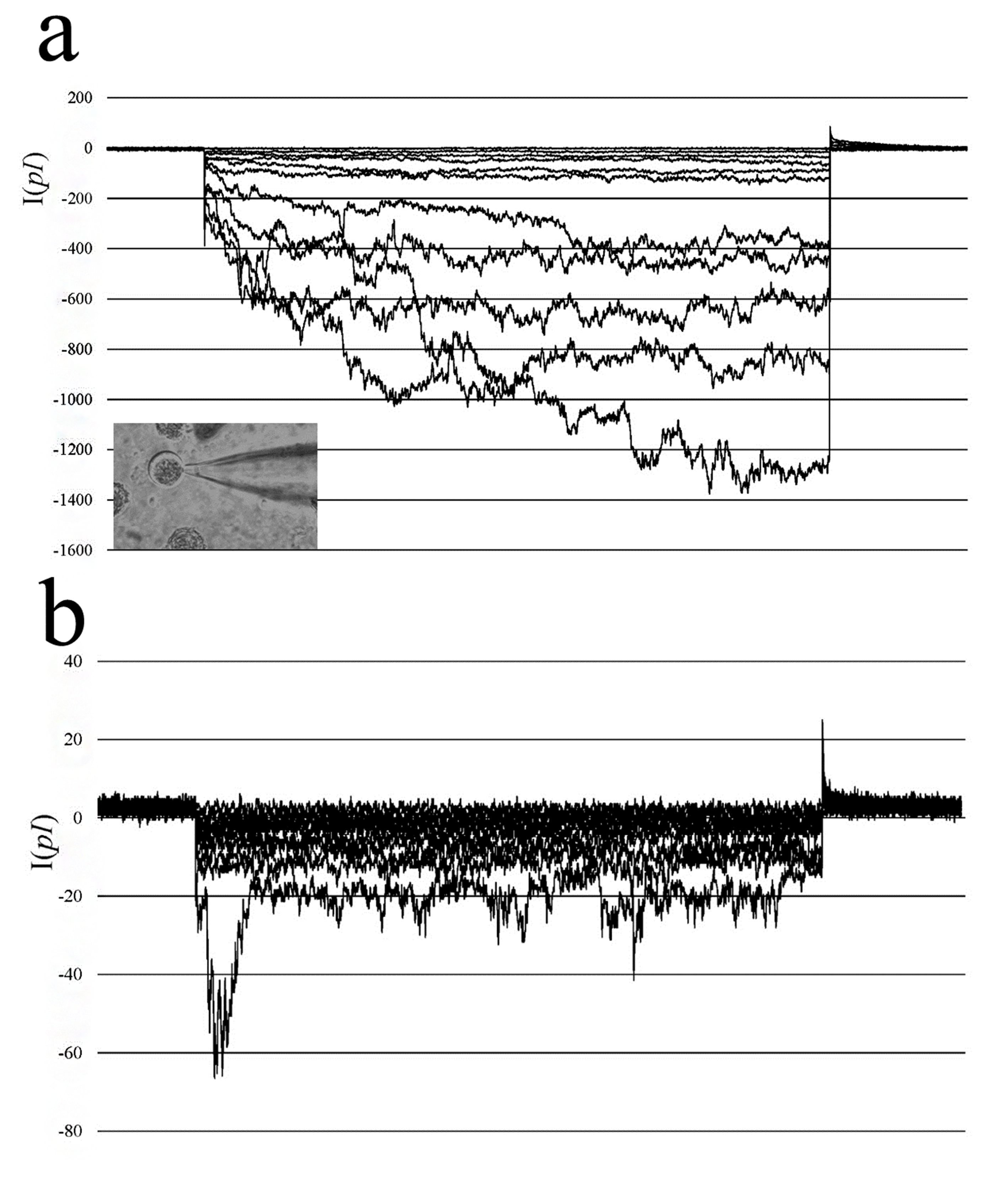

Supplement: S3 Fig — Cd2+ is widely used as an effective Ca2+ channel blocker in both animal and plant cells. As shown in (a) and (b), 50 μM Cd2+ markedly inhibited hyperpolarization-activated inward currents (n = 5). (a) Normal Ca2+ currents recorded in 10 mM extracellular Ca2+ (control). The inset shows a pipette forming a giga seal with a protoplast membrane of the pollen tube apical region. Giga seal resistances were all greater than 1 GΩ. (b) Ca2+ currents with 50 μM Cd2+ added to the bath solution. (TIF) [file pone.0152320.s003.tif]

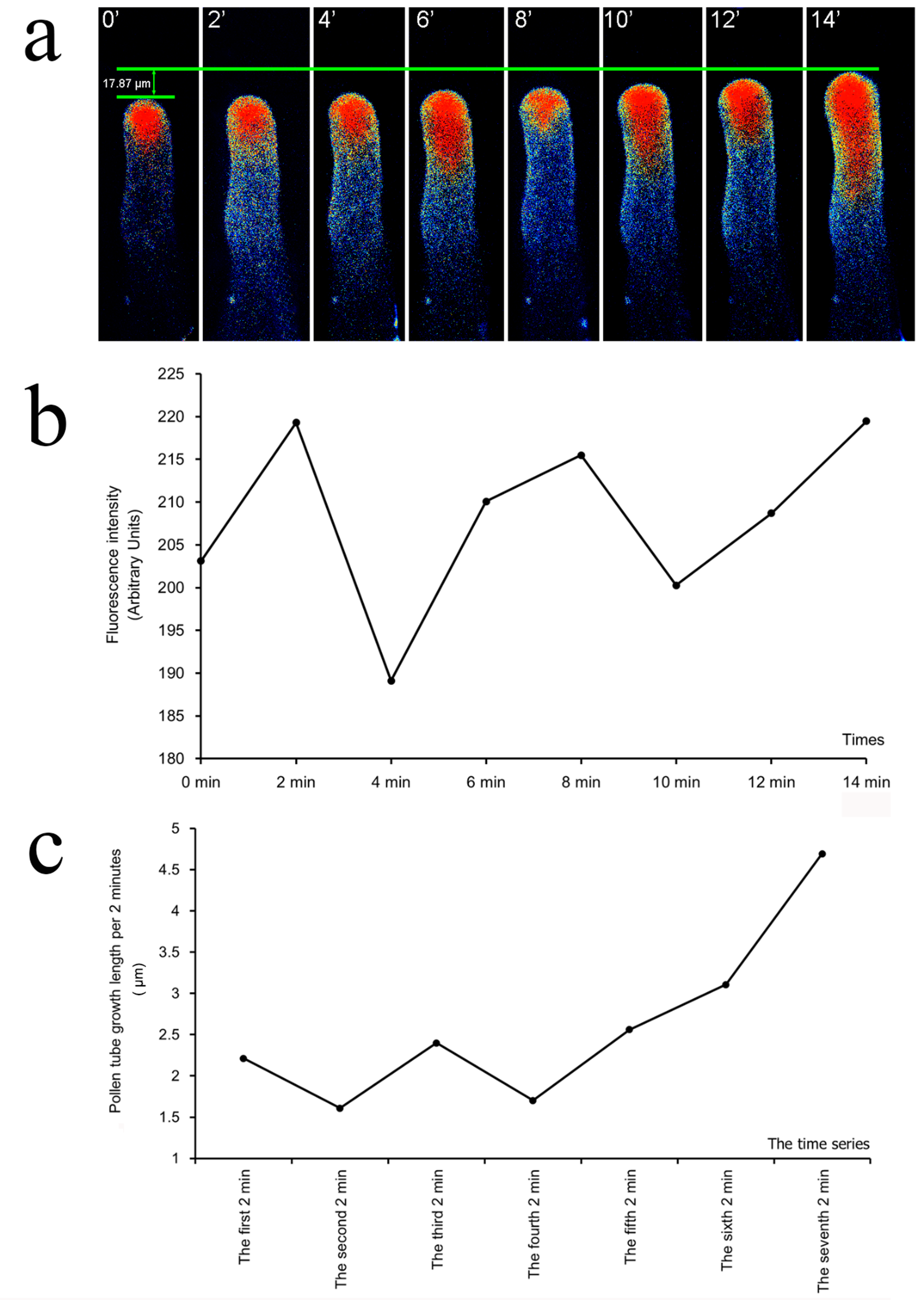

Supplement: S4 Fig — Pollen tubes maintained Ca2+ gradients at the apex pollen tube for 15 min during normal growth (a-b). Ca2+ concentration fluctuation at the tip of the pollen tube during pollen tube growth. (c) Growth of the pollen tube length every two minutes. The intensity of the red color indicates higher concentrations of calcium ions. (TIF) [file pone.0152320.s004.tif]

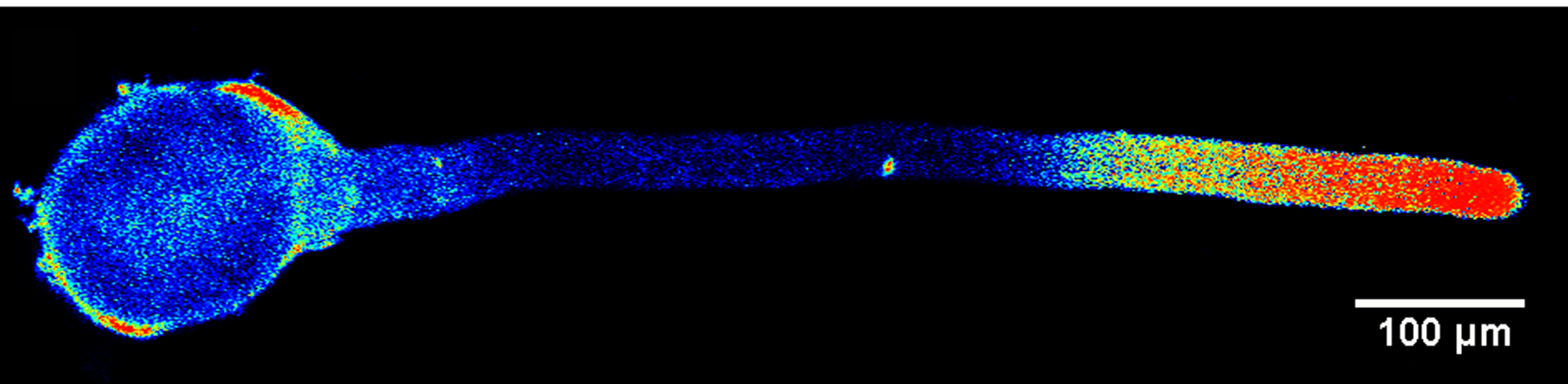

Supplement: S5 Fig — Pollen tubes loaded with fluo-4/AM along with cell lysis solution for 15 min. (TIF) [file pone.0152320.s005.tif]

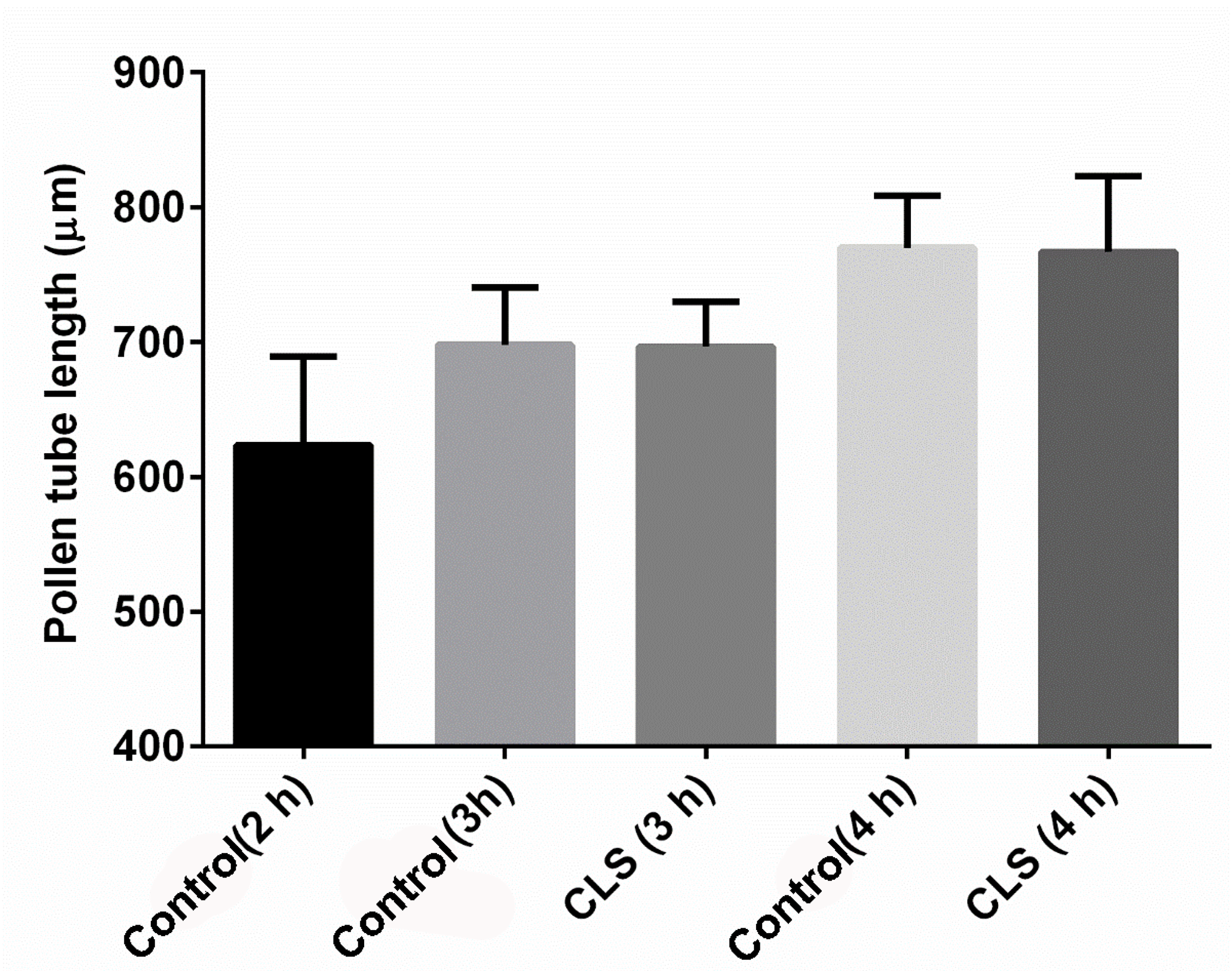

Supplement: S6 Fig — After 15 min, the pollen was washed three times and the culturing was continued. No difference in pollen tube length was observed between the treatment and control at 3 h and 4 h. This result suggests that the pollen tube activity was unaffected by a brief (15 min) cell lysis solution treatment. More than 50 growing pollen tubes were quantified per line. Error bars represent ± SD. CLS: cell lysis solution. (TIF) [file pone.0152320.s006.tif]
